# Peripheral inflammation mediates the relationship between early life stress and alcohol use

**DOI:** 10.1007/s00213-026-07009-8

**Published:** 2026-01-28

**Authors:** Kaitlin R. McManus, Craig Enders, Dylan E. Kirsch, Malia Belnap, Erica N. Grodin, Lara A. Ray

**Affiliations:** 1 Department of Psychology, University of California, Los Angeles, 1285 Franz Hall, Box 951563, Los Angeles, CA 90095-1563 USA; 2 Neuroscience Interdepartmental Program, University of California at Los Angeles, Los Angeles, CA 90095 USA; 3 Department of Psychiatry and Biobehavioral Sciences, University of California, Los Angeles, CA 90095 USA; 4 Brain Research Institute, University of California, Los Angeles, Los Angeles, CA 90095 USA

**Keywords:** Early life stress, Alcohol use, Alcohol use disorder, Inflammation, Sex differences

## Abstract

**Rationale:**

Early life stress (ELS) is associated with an increased risk for alcohol consumption and development of alcohol use disorder (AUD). Inflammation may be a sex-dependent link between ELS and alcohol use; however, there is limited evidence to support such an association in individuals with AUD.

**Methods:**

This secondary analysis utilized cross-sectional mediation to investigate the role of peripheral inflammation in the relationship between ELS and alcohol consumption (i.e., drinks per drinking day (DPDD)) in treatment-seeking individuals with AUD (*n* = 99; 60 M/39 F). A cross-sectional moderated mediation analysis was conducted to test sex as a moderator in the link between ELS and inflammation for the above relationship. ELS was conceptualized as a dichotomous “no-moderate ELS” vs. “high-ELS” predictor.

**Results:**

High-ELS was associated with higher inflammation (95%CI 0.039,1.558), and higher inflammation was associated with greater DPDD *(*95%CI 0.024,0.098). A mediation effect emerged where high-ELS influenced greater DPDD through elevated inflammation (*ab* = 0.044, 95%CI 0.002,0.113). No direct effects between ELS and DPDD emerged (95%CI -0.177,0.108). The interaction between ELS and sex on inflammation was significant for females (95%CI: 0.304,2.719) but not males (95%CI: -0.634,1.270). The mediation relationship between ELS and DPDD through inflammation was significant for females (*ab(female)* = 0.087, 95%CI: 0.013,0.199) but not males (95%CI: -0.040,0.086).

**Conclusions:**

Findings suggest that ELS is associated with recent alcohol use through peripheral inflammation in treatment-seeking individuals with AUD and indicate that this pathway is sex-dependent. Inflammation may be a treatment target for individuals with AUD who have experienced ELS, especially females. Clinical Trials Registration: NCT03594435, registered July 11, 2018.

## Introduction

Early life stress (ELS) is a risk factor for alcohol use disorder (AUD; (Greenfield et al. 2002; Kirsch et al. 2020). While efforts have been made to elucidate the association between ELS and AUD, these links are often *correlational in nature* and have not examined their impact on *clinical outcomes* for AUD (D. E. Kirsch, Grodin, Gillis, Kirsch et al. 2024a, b; D. E. Kirsch, Grodin, Nieto, Kirsch et al. 2024a, b), such as alcohol consumption. Moreover, it remains unclear *how* this risk factor may lead to clinical outcomes for AUD. Therefore, the present study examined peripheral inflammation as a potential mechanism through which ELS is associated with alcohol consumption in treatment-seeking adults with AUD and examined sex as a moderator of this mechanism.

ELS, or early life exposure to psychological stress and trauma (i.e., abuse, neglect, dysfunction), increases risk for AUD in adulthood (Capusan et al. 2021; Dube et al. 2002). Greater levels of trauma may be associated with greater alcohol-related negative consequences (Felitti et al. 1998; Hallihan et al. 2023), including earlier age of AUD onset (Kirsch et al. 2020), transition to more severe AUD (Greenfield et al. 2002), and less AUD treatment responsiveness (Schückher et al. 2019). ELS has been measured through the Adverse Childhood Experiences (ACE) questionnaire (Felitti et al. 2014), where evidence suggests that individuals who experience four or more ACEs have a 7.2-fold greater risk for developing AUD (Anda et al. 2006). Risk for heavy alcohol use is higher in females who have experienced ELS in comparison to males who have experienced ELS (Cronin et al. 2016; Heffner et al. 2011). Given the correlational links associating ELS with AUD and the potential sex-dependent nature of these links, it is important to consider mechanistically how ELS may lead to drinking behaviors associated with AUD. Identifying the pathway through which ELS confers risk for AUD symptomatology can help support targeted treatment development.

One proposed mechanism for ELS driving alcohol use is inflammation. ELS has been associated with increased peripheral inflammation in people who drink alcohol and those with AUD (Battista et al. 2023; Kirsch et al. 2024a; D. E. Kirsch, Grodin, Nieto, Kirsch et al. 2024a, b; Raposa et al. 2014). Specifically, high-ELS exposure (i.e., score of 4 + on ACEs questionnaire) has been associated with increased peripheral inflammation and greater negative emotionality, a facet of the AUD profile (D. E. Kirsch, Grodin, Nieto, Kirsch et al. 2024a, b). The inflammatory effects of ELS may be sex-dependent (Baldwin et al. 2018), where females may be more susceptible to the biological effects of ELS compared to males (Mengelkoch and Slavich 2024). Indeed, within a population of individuals with AUD, females with AUD and high-ELS exposure have higher c-reactive protein (CRP) levels, as well as higher composite scores of peripheral inflammatory markers, compared to males with AUD and high-ELS (Kirsch et al. 2024; D. E. Kirsch, Grodin, Nieto, Kirsch et al. 2024a, b). Further, the relationship between alcohol use and inflammation has been studied through preclinical and clinical research. Evidence suggests positive associations between chronic alcohol use and CRP (Alho et al., 2004) as well as inflammatory cytokines interleukin (IL)−6, IL-8, and tumor necrosis factor alpha (TNF-α; (Achur et al. 2010; Heberlein et al. 2014; Hillmer et al. 2020). Preclinical literature has utilized experimental inflammatory challenge paradigms to suggest that inducing inflammation drives alcohol consumption (Blednov et al. 2011; Decker Ramirez et al. 2023). Moreover, experimentally inhibiting inflammatory mechanisms has resulted in female mice consuming less alcohol than male mice, suggesting that female mice are more sensitive to inflammation-driven alcohol use behaviors than male mice (Blednov et al. 2005; Lowe et al. 2020). Therefore, ELS may increase inflammation in a sex-dependent manner, which then drives alcohol use. In sum, mechanistic research linking ELS with clinical alcohol use outcomes is sparse as previous associations are *correlational in nature*. Though inflammation is a promising shared mechanism that links ELS and alcohol use, we currently lack a well-replicated, distinct, biological mechanism driving the relationship between ELS and alcohol use.

To our knowledge, one study to date has conducted mediation analyses to identify CRP as a mechanism linking ELS to alcohol use in a clinical sample of adults with and without AUD (Battista et al. 2023). Findings suggested that elevated CRP mediated the relationship between ELS and increased alcohol consumption in a sample of non-treatment-seeking individuals with AUD. This line of research should be replicated and extended into a clinical AUD sample that considers inflammatory markers beyond CRP as well as the moderating role of sex in the relationship between ELS, inflammation, and alcohol use.

Therefore, the present study sought to clarify the link between ELS, peripheral inflammation, and recent alcohol use in treatment-seeking individuals with AUD. Specifically, our primary objectives were to (1) extend previous findings that inflammation mediates the relationship between ELS and alcohol use (Battista et al. 2023) in a sample of treatment-seeking individuals with moderate-severe AUD; (2) utilize a broader measurement of inflammation that includes inflammatory cytokines by creating a composite of inflammatory markers; and (3) test whether sex, defined as biological sex assigned at birth, moderated the relationship between ELS and inflammation, which would thereby indicate that inflammation is a sex-dependent cross-sectional mediator in the relationship between ELS and alcohol use.

## Methods

### Data source and sample

This secondary analysis culled data from a clinical pharmacotherapy study conducted in the UCLA Addictions Laboratory from July 2018 through January 2023 (ClinicalTrials.gov identifier: NCT03594435, registered July 11, 2018; (Ray et al. 2025). The pharmacotherapy study was investigating the use of ibudilast, a neuroimmune modulator, to treat individuals with AUD. The UCLA Institutional Review Board approved all study procedures, and all study participants provided written informed consent prior to completing study procedures. Participants were recruited from the greater Los Angeles area through online, newspaper, and mass transit advertisements, as well as targeted recruitment through a database of previous participants willing to be recontacted for future studies. All demographic, clinical, and inflammatory marker data used in the present analysis were collected *prior* to any medication administration procedures thereby providing an unbiased assessment of inflammatory load.

Participants were a community sample of treatment-seeking individuals with moderate-severe AUD. All participants completed initial screening procedures via phone to assess preliminary eligibility followed by in-person formal screening procedures. Inclusion criteria were as follows: (1) ages 18–65, (2) current (past 12-months) DSM-5 diagnosis of current moderate or severe AUD as determined by the clinician-administered Structured Clinical Interview for DSM-5 (SCID; (First et al. 2015), and (3) treatment-seeking for AUD. Exclusion criteria were as follows: (1) urine screen positive for all drugs of misuse besides cannabis, (2) breath alcohol concentration of > 0.00 g/dl, (3) current acute withdrawal symptoms as indicated by Clinical Institute Withdrawal Assessment for Alcohol Withdrawal (CIWA-Ar; (Sullivan et al. 1989) score *≥* 10, (4) active COVID or sickness symptom (i.e., fever) at blood draw, (5) if female: pregnant, nursing, or refusal to use reliable method of birth control, (6) a medical condition that may interfere with safe study participation, (7) current (past year) serious suicidal intention/plan and/or past suicide attempt, (8) current use of prescription medication that contraindicates use of ibudilast, (9) current use of medications for AUD, (10) current use of psychotropic medications except for stable (i.e., *≥* 4 week use) antidepressants, and (11) AST, ALT, and GGT levels > 3 times the normal limit.

### Procedures

***Measures*** Participants completed questionnaires at baseline, prior to medication randomization. Participants completed the Alcohol Use Disorders Identification Test (AUDIT; (Saunders et al. 1993) as a measure of alcohol use severity. Participants completed the Adverse Childhood Experience Questionnaire, which is a 10-item yes/no self-report assessment to assess for abuse, neglect, and household dysfunction experienced during childhood (Felitti et al. 1998). ACE score was transformed into a dichotomous indicator of ELS. Specifically, in line with our previous work, ACE scores of 0–3 were categorized as “no-moderate ELS” and ACE scores of 4 + were categorized as “high-ELS” to investigate ELS as a dichotomous predictor (Kirsch et al. 2024; D. E. Kirsch, Grodin, Nieto, Kirsch et al. 2024a, b). At the medication randomization visit, prior to medication titration, a Timeline Follow Back (TLFB; (Sobell and Sobell 1992) interview-based assessment was used to capture alcohol, tobacco, and cannabis use since the baseline visit. Past week drinks per drinking day was calculated based off the TLFB to provide an estimate of recent alcohol use at the time of blood collection. Importantly, the TLFB assessment and blood collection were performed at the same study visit, thereby meaning that the retrospective assessment of alcohol use preceded the blood collection. As such, the present mediation analyses are cross-sectional in nature and causal temporal conclusions cannot be drawn.

***Circulating plasma cytokines and C-reactive protein*** Blood samples were collected at the randomization visit prior to medication titration to measure circulating levels of CRP and peripheral cytokines TNF-α, IL-6, IL-8, IL-10, and interferon gamma (IFN-γ). Specifically, whole blood samples were collected in ethylenediaminetetraacetic acid (EDTA) tubes and were centrifuged in a refrigerated centrifuge at 4 °C to obtain plasma. Plasma was aliquoted and stored in a −80 °C freezer for batch testing. CRP levels were measured using the high-sensitivity Human CRP Quantikine ELISA (R&D Systems) according to the manufacturer’s protocol. Samples were assayed in duplicate, and a lower limit of detection of 0.2 milligrams per liter (mg/L) was utilized (Grodin et al. 2023). The mean inter-assay coefficient of variation (CV) was 5.7% and the mean instar-assay CV was 3.3%. For the samples (8%, *n* = 8) with CRP levels below the limit of detection (0.2 mg/L), a value of 0.2 mg/L was assigned.

Peripheral cytokines were assessed using the Meso Scale Discovery MULTI-SPOT Assay System (Rockville, MD) according to the manufacturer’s protocol. Plasma samples were assayed in duplicate on a custom 5-plex from the Proinflammatory Panel 1 Human Kit. Electrochemiluminescence (ECL) signals were measured on the MESO QuickPlexSQ 120 (Rockville, MD). The mean intra-assay CV for TNF-α was 7.6%, and the mean inter-assay CV < 12%, with similar or lower CV’s for other inflammatory markers. TNF-α, IL-8, IL-10, and IFN-γ levels were detectable in all subjects. For IL-6, 5% of the samples (*n* = 5) were below the level of detection (0.2 picograms per milliliter (pg/mL)) and were assigned a value of 0.2 pg/mL.

### Data analysis

We sought to create an inflammation composite score to capture systemic inflammation (Kuhlman et al. 2024). Concentrations for each pro-inflammatory cytokine (TNF-α, IL-6, IL-8, IL-10, IFN-γ) and CRP were log-transformed due to their expected non-normal distributions. Following, Pearson correlations were run to assess the relationships between inflammatory markers. Strong associations were considered to have Pearson correlation coefficients *≥* 0.30 (Mukaka 2012). These log-transformed markers were then converted to z-scores, and the z-scores were subsequently summed to create the inflammation composite.

Three markers emerged as being strongly positively associated with one another (i.e., r ≥ 0.30). These included log CRP, log IL-6, and log TNF- α. Specifically, log CRP was significantly correlated with log IL-6 (*r* = 0.46) and log TNF-α (*r* = 0.34), while log IL-6 and log TNF- α were significantly correlated with one another (*r* = 0.30). No other markers of inflammation (IL-8, IL-10, and IFN-γ) were significantly strongly correlated with all three markers (*r*s < 0.30); additional correlations between markers of inflammation can be found in Table 1. Therefore, log CRP, log IL-6, and log TNF-α were z-score transformed and summed together to form the inflammation composite. The inflammation composite was positively and significantly associated with alcohol use (i.e., DPDD; *r* = 0.33, *p* < 0.001) and early life stress (i.e., ACEs sum score; *r* = 0.22, *p* = 0.028).

**Table 1:** Correlations between markers of inflammation

	Log CRP	Log IL-6	Log IL-8	Log TNF-α	Log IFN-γ	Log IL-10
**Log CRP**	1.00	**0.46**	0.08	**0.34**	0.18	0.16
**Log IL-6**	**0.46**	1.00	0.11	**0.30**	0.33	0.10
**Log IL-8**	0.08	0.11	1.00	0.31	0.13	0.18
**Log TNF-α**	**0.34**	**0.30**	0.31	1.00	0.54	0.38
**Log IFN-γ**	0.18	0.33	0.13	0.54	1.00	0.25
**Log IL-10**	0.16	0.10	0.18	0.38	0.25	1.00

*CRP * C-reactive protein, *IL* interleukin, *TNF-*α *tumor* necrosis factor alpha, *IFN*-*γ* interferon gamma. Bolded correlation coefficient values indicate strongly positive associations between inflammatory markers retained in inflammation composite

**Fig. 1:**
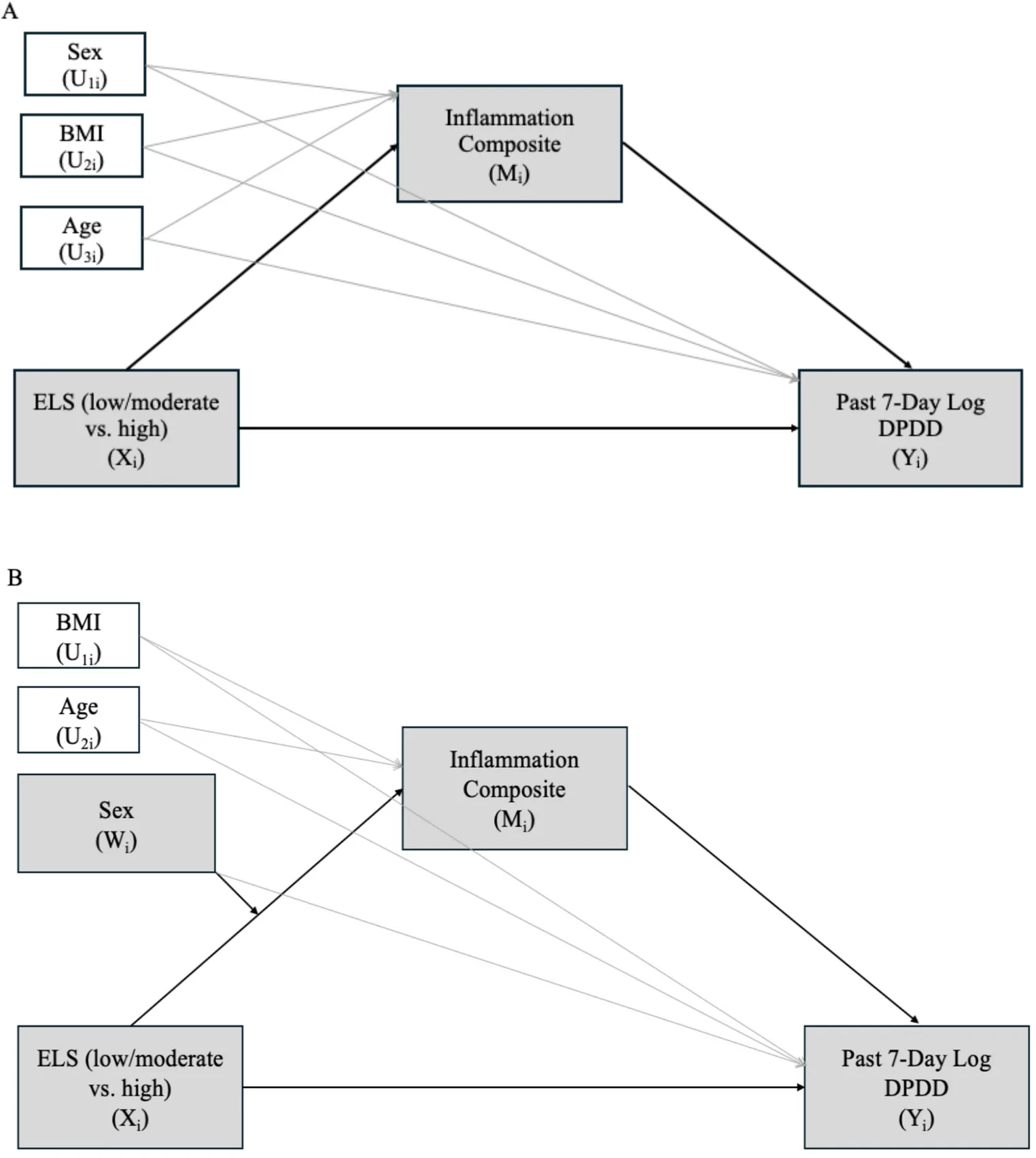
(A) Path diagram of mediation analysis. Inflammation composite is proposed mediator between early life stress (ELS) predictor and past 7-day log drinks per drinking day (DPDD) outcome. Sex, body mass index (BMI) and age are covariates on mediator and outcome. (B) Path diagram of moderated mediation analysis. Sex is proposed moderator of path between ELS predictor and inflammation composite mediator. BMI and age are covariates on mediator and outcome; sex is a covariate on outcome

A cross-sectional mediation analysis tested the inflammation composite as a potential mediator between ELS and alcohol use. Specifically, a single mediation model was run to test dichotomous ELS score as a predictor of past 7-day alcohol use (drinks per drinking day (DPDD); (Agarwal et al. 2025; Hoeppner et al. 2010), with the composite inflammation score as the potential mediator. Past 7-day DPDD was log transformed due to its non-normal distribution. A constant was added to each DPDD value prior to log transforming to avoid undefined values of negative infinity. BMI, age, and sex were included as covariates in both the mediator and outcome components of the mediation model (see Fig. 1A) given their known effects on inflammation (Liqiang et al. 2023).

A cross-sectional moderated mediation analysis was subsequently run to test sex as a potential moderator of the relationship between the ELS predictor and inflammation mediatory, with past 7-day log-DPDD as the outcome variable. Sex-specific conditional indirect effects were derived by computing the product of coefficients separately for males and females. BMI and age were used as covariates in both the mediator and outcome components of the moderated mediation model, while sex was used as a covariate in the outcome component (see Fig. 1B).

Sensitivity analyses were run using a winsorization method to transform high levels of inflammatory cytokines and CRP. Specifically, high levels were defined as values greater than (mean cytokine or CRP + 2*standard deviation cytokine or CRP). Inflammatory marker levels above these thresholds were transformed to be equivalent to the calculated threshold. These markers were not winsorized in the main analyses because our rigorous blood processing procedures ensure reliability and validity of inflammatory markers, thereby indicating that high inflammatory marker values are feasible and interpretable. However, to ensure that the models were not being driven by individuals with high levels of inflammatory markers, sensitivity analyses were conducted transforming high levels for said individuals. Moreover, given that the cross-sectional mediation analysis design precludes the determination of a temporal, causal, association between inflammation and alcohol use, additional sensitivity analyses were conducted to provide support for ELS influencing alcohol use through inflammation. Specifically, the inverse association was probed to examine if ELS influences inflammation through alcohol use. A cross-sectional mediation analysis was conducted to assess past 7-day log DPDD as a mediator between dichotomous ELS score (no/moderate ELS, high-ELS) and the inflammation composite while controlling for age, BMI, and sex in both the mediator and outcome parts of the model.

All analyses were conducted in RStudio (Posit team 2025) using Bayesian Markov chain Monte Carlo (MCMC) estimation in Blimp 3 (Keller and Enders 2021). In both the mediation and moderated mediation models, two MCMC chains with random starting values were run to generate posterior distributions consisting of 10,000 iterations retained after an initial 10,000-iteration burn-in period. The component path coefficients, derived from regression slopes, were multiplied at each of the 10,000 iterations to form an estimate of the product of coefficients. A posterior distribution of plausible indirect effects was formed by the accumulation of product coefficient estimates. MCMC estimation distributions of indirect effects are not constrained to a particular form and can be non-normal. Therefore, MCMC estimation results in appropriate asymmetric 95% intervals for significance testing (Enders 2022). Significance testing was achieved with 95% Bayesian credible intervals, where significance was indicated if the posterior distribution of the conditional indirect effect did not include 0.

## Results

### Sample characteristics

The final sample consisted of 99 (60 M/39 F) treatment-seeking individuals with moderate to severe AUD. In terms of alcohol use characteristics, participants endorsed an average of six symptoms of AUD on the SCID assessment, with 66 individuals meeting criteria for severe AUD. Participants had an average AUDIT score of 19.99 (SD = 7.29), consumed an average of 7.47 DPDD (SD = 5.39) in the 30 days prior to study enrollment, and an average of 5.79 DPDD (SD = 3.90) in the 7 days prior to blood draw for inflammatory markers. Past 7-day DPDD was significantly positively correlated with past 30-day DPDD (*r* = 0.41, *p* < 0.001), providing support for using the 7-day DPDD outcome in all analyses. In terms of additional clinical characteristics, participants endorsed experiencing an average of 3.29 (SD = 2.38) ACEs, with 60 participants meeting the threshold for no/moderate-ELS and 39 participants meeting the threshold for high-ELS. Participants between ELS groups did not significantly differ on age, sex, race, or ethnicity (all *p*s > 0.05). Full sample characteristics can be found in Table 2.

**Table 2:** Sample demographic and clinical characteristics

Variable	Total Sample	No/Mod-ELS	High-ELS	ELS Group
	*N* = 99	*N* = 60	*N* = 39	p-value
**Demographic Characteristics**				
Age	44.30 *±* 10.95	44.40 *±* 10.58	44.15 *±* 11.64	0.914
Sex (no., %)				0.835
Male	60 (60.61)	37 (61.67)	23 (58.97)	
Female	39 (39.39)	23 (38.33)	16 (41.03)	
Race (no., %)				0.097
White	49 (49.49)	34 (56.67)	15 (38.46)	
Black/African American	24 (24.24)	13 (21.67)	11 (28.21)	
American Indian/Alaskan Native	3 (3.03)	1 (1.67)	2 (5.13)	
Asian	1 (1.01)	1 (1.67)	0 (0.00)	
Pacific Islander	2 (2.02)	2 (3.33)	0 (0.00)	
Mixed Race	13 (13.13)	4 (6.67)	9 (23.08)	
Other	7 (7.07)	5 (8.33)	2 (5.13)	
Hispanic/Latinx (no., %)				0.825
Yes	31 (31.31)	18 (30.00)	13 (33.33)	
No	68 (68.69)	42 (70.00)	26 (66.67)	
Years of Education	14.33 *±* 3.30	14.73 *±* 3.46	13.71 *±* 2.98	0.133
Body Mass Index	27.93 *±* 5.92	27.57 *±* 6.20	28.49 *±* 5.51	0.456
BDI-II Symptom Count	7.52 *±* 6.85	7.60 *±* 7.34	7.38 *±* 6.09	0.879
ACEs	3.29 *±* 2.38	1.67 *±* 0.97	5.79 *±* 1.59	**< 0.001**
**Inflammation Characteristics**				
CRP mg/L	3.90 *±* 8.64	2.19 *±* 2.58	6.53 *±* 13.06	**0.014**
IFN-γ pg/mL	13.53 *±* 37.23	8.52 *±* 7.67	21.25 *±* 58.16	0.097
TNF-α pg/mL	0.82 *±* 0.27	0.80 *±* 0.24	0.84 *±* 0.32	0.425
IL-6 pg/mL	0.89 *±* 1.54	0.88 *±* 1.78	0.92 *±* 1.09	0.902
IL-8 pg/mL	4.71 *±* 3.64	4.03 *±* 2.26	5.76 *±* 4.93	**0.020**
**Drinking Characteristics**				
Past 30-day Total Drinks	157.20 *±* 105.90	149.99 *±* 99.31	169.30 *±* 115.80	0.400
Past 7-day DPDD	6.22 *±* 3.69	5.63 *±* 3.65	6.02 *±* 4.30	0.630
AUDIT	19.99 *±* 7.29	19.00 *±* 7.00	21.00 *±* 7.83	0.118
CIWA-Ar	0.82 *±* 1.53	0.77 *±* 1.41	0.87 *±* 1.73	0.750
SCID AUD Symptom Count	6.66 *±* 1.89	6.27 *±* 1.60	7.26 *±* 2.16	**0.010**
AUD Diagnosis (no., %)				0.513
Mild	0.00 (0.00)	0.00 (0.00)	0.00 (0.00)	
Moderate	32 (32.65)	21 (35.00)	11 (28.21)	
Severe	66 (67.35)	38 (63.00)	28 (71.79)	
**Other**				
Inflammatory or anti-inflammatory medication use	17 (17.17)	9 (15.00)	8 (20.51)	0.477
Recent sickness	8 (8.08)	5 (8.33)	3 (7.69)	0.909
THC Positive	15 (15.15)	8 (13.33)	7 (17.95)	0.531

*ELS* early life stress, *BDI-II* Beck Depression Inventory-II, *ACEs* Adverse Childhood Experiences, *CRP* C-reactive protein, *IFN-γ* interferon gamma, *TNF-α* tumor necrosis factor alpha, *IL* interleukin, *DPDD* drinks per drinking day, *AUDIT* alcohol use disorder identification test, *CIWA-Ar* Clinical Institute Withdrawal Assessment for Alcohol Withdrawal, *SCID* Structured Clinical Interview for DSM-5, *AUD* alcohol use disorder, *THC* tetrahydrocannabinol. Bolded p-values indicate statistically significant differences between ELS groups

### Mediation model

**Fig. 2:**
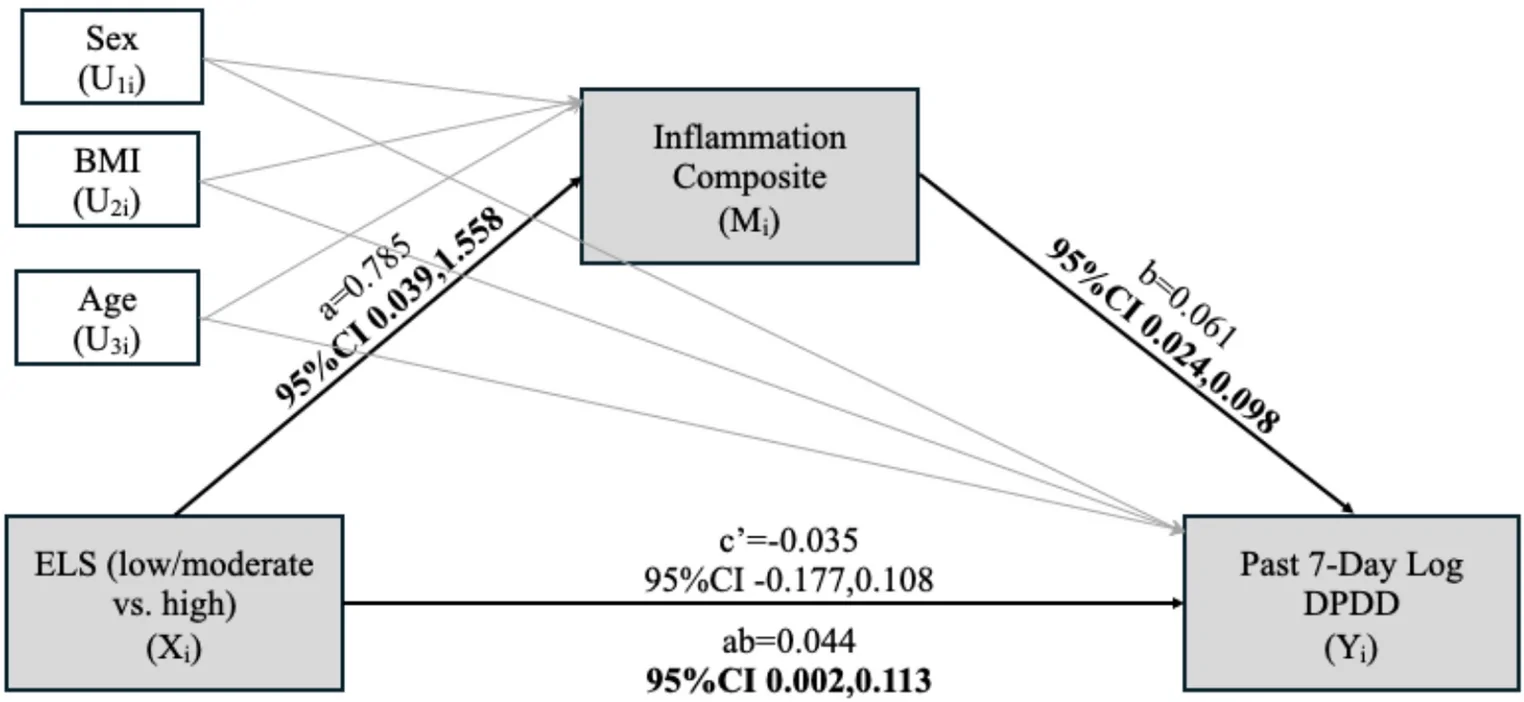
Mediation model assessing whether peripheral inflammation mediates the association between early life stress (ELS; low/moderate vs. high) and recent alcohol consumption (past 7-day log-drinks per drinking day (DPDD)), controlling for sex, body mass index (BMI), and age on both the mediator and outcome. Results indicate that peripheral inflammation mediated the relationship between ELS and DPDD. Bolded values indicate significance

A cross-sectional mediation analysis was conducted to assess the inflammation composite as a mediator between dichotomous ELS score (no/moderate ELS, high-ELS) and past 7-day log DPDD while controlling for age, BMI, and sex in both the mediator and outcome parts of the model. ELS was significantly positively associated with the inflammation composite (*a* = 0.785, SD = 0.39, 95%CI 0.039,1.558), where high-ELS was associated with higher levels of inflammation. The inflammation composite in turn was significantly positively associated with past 7-day log DPDD (*b* = 0.061, SD = 0.02, 95%CI 0.024,0.098), where higher levels of inflammation predicted higher DPDD. The direct effect of ELS score on DPDD was not significant when accounting for the inflammation composite mediator (*c’* =−0.035, SD = 0.07, 95%CI −0.177,0.108). However, the indirect effect of ELS score on DPDD through the inflammation mediator was significant (*ab* = 0.044, SD = 0.030, 95%CI 0.002,0.113), where high-ELS predicted higher levels of inflammation, which in turn predicted higher DPDD. Therefore, results suggest a mediation effect of dichotomous ELS score on DPDD through inflammation (see Fig. 2).

## Moderated mediation model

A cross-sectional moderated mediation analysis was conducted to assess if the relationship between dichotomous ELS score and past 7-day log DPDD through the inflammation composite was moderated by sex. Age and BMI were controlled for in both the mediator and outcome parts of the model, while sex was controlled for in the outcome of the model. Sex was tested as a moderator on the pathway between ELS and the inflammation composite. The interaction between ELS and sex on the inflammation composite was significant, where the relation between ELS and inflammation was significant and positive for females (*a*(*female*) = 1.51, SD = 0.61, 95%CI: 0.304,2.719) but not for males (*a*(*male*) = 0.32, SD = 0.49, 95%CI: −0.634,1.270). Moreover, the indirect effect, or relationship between ELS and past 7-day log DPDD through the inflammation composite, was significant for females (*ab(female)* = 0.087, SD = 0.05, 95%CI: 0.013,0.199) but not for males (*ab(male)* = 0.017, SD = 0.031, 95%CI: −0.040,0.086). Specifically, in females, high-ELS predicted higher levels of inflammation, which in turn predicted higher DPDD. The index of moderated mediation, or the difference in indirect effects between females and males, was not significant (95%CI: −0.022,0.194).

### Sensitivity analyses

***Mediation model*** Following winsorization, the dichotomous ELS score was significantly associated with the inflammation composite (*a* = 0.775, SD = 0.38, 95%CI 0.043,1.533). The inflammation composite in turn was associated with past 7-day log DPDD (*b* = 0.065, SD = 0.02, 95%CI 0.028,0.103). The direct effect of dichotomous ELS score on DPDD was not significant when accounting for the inflammation composite mediator (*c’*=−0.038, SD = 0.07., 95%CI −0.179,0.104). However, the indirect effect of ELS score on DPDD through the inflammation mediator was significant (*ab* = 0.047, SD = 0.03, 95%CI 0.002,0.117). Therefore, sensitivity analyses using winsorization supported the main results that suggest a mediation effect of dichotomous ELS score on DPDD through inflammation.

***Moderated mediation model*** Following winsorization, a moderated mediation analysis was conducted to assess if the relationship between dichotomous ELS score and past 7-day log DPDD through the inflammation composite was moderated by sex while controlling for age and BMI. The interaction between ELS and sex on the inflammation composite was significant for females (*a*(*female*) = 1.38, SD = 0.60, 95%CI: 0.196,2.574) but not for males (*a*(*male*) = 0.38, SD = 0.48, 95%CI: −0.554,1.320). The relationship between ELS and past 7-day log DPDD through the inflammation was significant for females (*ab(female)* = 0.086, SD = 0.05, 95%CI: 0.010,0.198) but not for males (*ab(male)* = 0.023, SD = 0.03, 95%CI: −0.037,0.096). Therefore, the sensitivity analyses support the main moderated mediation results.

***Alcohol use as mediator*** The cross-sectional mediation analysis design precludes the determination of a temporal, causal, association between inflammation and alcohol use. However, to provide support for ELS influencing alcohol use through inflammation, the inverse association was probed to examine if ELS influences inflammation through recent alcohol use. A cross-sectional mediation analysis was conducted to assess past 7-day log DPDD as a mediator between dichotomous ELS (no/moderate ELS, high-ELS) and the inflammation composite while controlling for age, BMI, and sex in both the mediator and outcome parts of the model. ELS was not significantly associated with the past 7-day log DPDD (*a* = 0.012, SD = 0.07, 95%CI −0.129,0.159). Past 7-day log DPDD was significantly positively associated with the inflammation composite (*b* = 1.706, SD = 0.53, 95%CI 0.652,2.749), where higher DPDD predicted higher levels of inflammation. The direct effect of ELS on the inflammation composite was significant when accounting for the alcohol use mediator (*c’ *= 0.039, SD = 0.37, 95%CI 0.039,1.497). However, the indirect effect of ELS on inflammation through the DPDD mediator was not significant (*ab* = 0.018, SD = 0.13, 95%CI −0.242,0.296). Therefore, results suggest that higher ELS predicts higher levels of inflammation, and alcohol use predicts higher levels of inflammation. However, results suggest that these pathways are independent of one another as ELS does not predict inflammation through alcohol use. Therefore, sensitivity analyses probing the inverse association of ELS influencing alcohol use through inflammation revealed a null indirect effect of ELS score on inflammation through the DPDD mediator, thereby providing support that ELS predicts alcohol use through inflammation, but does not predict inflammation through alcohol use. Given that a mediation relationship between ELS, alcohol use, and inflammation was not identified, a moderated mediation model was not conducted to examine if sex moderated the relationship between dichotomous ELS score and inflammation through past 7-day log DPDD.

## Discussion

The present study examined the relationships between ELS and alcohol consumption through peripheral inflammation. Findings indicate that high-ELS is associated with recent alcohol consumption through peripheral inflammation. Specifically, high levels of ELS (vs. no/moderate-ELS) were associated with higher levels of peripheral inflammation, which in turn predicted more recent alcohol consumption. Findings indicated a sex-dependent pattern in these findings, where the association between high-ELS with increased alcohol consumption through higher levels of peripheral inflammation was especially salient for females, but not males.

These findings extend the literature showing that greater levels of ELS are a risk factor for negative consequences (Felitti et al. 1998; Hallihan et al. 2023) such as alcohol use, as our results demonstrate that high-ELS was associated with more risk for recent alcohol use through peripheral inflammation compared to moderate (or no) ELS. This finding of high-ELS as a risk factor for alcohol use through inflammation is supported by preclinical literature that suggests ELS leads the immune system to be hyperactive in adulthood (Pinheiro et al. 2015; Viviani et al. 2014; Weber et al. 2017). This immune susceptibility has been indicated in translational human findings in which ELS leads to increased sensitivity to peripheral inflammation (Kuhlman et al. 2020) as well as potentiated inflammation in adulthood that increases risk for disease and death (Fagundes and Way 2014; Felitti et al. 1998). Our work extends the notion of ELS-induced immune susceptibility by suggesting that ELS-driven hyperactive inflammation in adulthood may contribute to alcohol consumption in individuals with AUD.

Prior literature has identified associations between ELS and increased risk for AUD in adulthood (Capusan et al. 2021; Dube et al. 2002) and between ELS and increased peripheral inflammation in individuals with AUD (Kirsch et al. 2024; D. E. Kirsch, Grodin, Nieto, Kirsch et al. 2024a, b; Raposa et al. 2014). Our study extends these findings by incorporating ELS, a composite of inflammatory markers, and alcohol use into a single model. There was no direct effect between ELS and alcohol consumption when controlling for peripheral inflammation, but the indirect effect between ELS, inflammation, and alcohol use was statistically significant. This pattern is consistent with mediation by peripheral inflammation. This finding suggests that the association between ELS and alcohol use may operate through a biological mechanism. Our model identified an indirect effect of 0.044, indicating that inflammation is likely only a part of the link between ELS and alcohol use. Proposed biological mechanisms for the relationship between ELS and alcohol use include altered brain connectivity (Agarwal et al. 2024) and DNA methylation (Bendre et al. 2019), which could additionally be bidirectionally related to inflammation. While our results underscore the importance of peripheral inflammation in driving the link between high-ELS and alcohol use, the causal mechanism behind ELS conferring risk for alcohol consumption is likely complex and multifaceted.

Interestingly, previous findings identified that the direct relationship between ELS and alcohol use was not fully dependent upon inflammation (Battista et al. 2023), such that the relationship held while controlling for inflammation. Our findings diverge from this previous study, as our results revealed a non-significant relationship between ELS and alcohol when controlling for inflammation. This divergence in findings may be due to differences in the sample characteristics, sample size, and measurement of inflammation. In terms of sample characteristics, our sample consisted of 99 treatment-seeking individuals with moderate-severe AUD. Battista and colleagues utilized a sample that consisted of 1,221 individuals with and without AUD. ELS increases risk for more severe AUD presentation (Greenfield et al. 2002), which was observed in our sample where individuals in the high-ELS group had significantly more symptoms of AUD compared to those in the no/moderate-ELS group. However, the overall presence of moderate-severe AUD diagnosis throughout our sample may have masked ELS-related differences in alcohol use. Indeed, within our sample there were no significant differences in past 7-day DPDD or past 30-day total alcohol drinks between ELS groups. As such, the moderate-severe AUD presentation may have led to high drinking patterns in both ELS groups, thereby reducing variability in the drinking outcome. Moreover, higher peripheral inflammation is associated with AUD and drinking outcomes (Achur et al. 2010; Heberlein et al. 2014). The presence of AUD throughout our sample and controlling for inflammation may have attenuated ELS-related differences in alcohol use, thereby leading to the lack of a significant direct effect between ELS and DPDD observed in our study. Lastly, in terms of inflammation measurement, Battista and colleagues measured inflammation with CRP, while our study integrated CRP with inflammatory cytokines to create an inflammation composite. CRP is considered a general systemic marker of inflammation, while inflammatory cytokines mediate inflammatory responses, including producing CRP (Del Giudice and Gangestad 2018). Therefore, inflammatory cytokines may be more sensitive markers of inflammation, while CRP is dependent on the signaling of cytokines. Results in the present study, therefore, may be capturing a broader extent of the mechanistic role of inflammation between ELS and alcohol consumption. Taken together, our work highlights the complex mechanistic function of inflammation as we show that both pro-inflammatory cytokines and CRP (via an inflammation composite) may drive the association between higher levels of ELS and higher levels of alcohol consumption in a treatment-seeking clinical sample of individuals with AUD.

Analyses revealed a significant interaction between ELS and female, but not male, sex on the inflammation mediator. Moreover, a pattern was observed where the conditional indirect effect of ELS on DPDD through inflammation was significant for females but not for males. Specifically, in females but not males, higher levels of ELS (vs. no/moderate ELS) were associated with higher levels of peripheral inflammation, which in turn predicted higher levels of recent alcohol consumption. This observed sex-dependent pattern aligns with previous work indicating that females may be more susceptible to the biological effects of ELS compared to males (Mengelkoch and Slavich 2024), where females with AUD and high-ELS have greater levels of peripheral inflammation compared to females with AUD and no/moderate ELS (Kirsch et al. 2024). Our work extends this finding by including a clinical outcome of alcohol consumption, where females with high-ELS had higher drinks per drinking day due to heightened levels of peripheral inflammation. While females were found to be susceptible to this pattern, males were not. Epidemiological studies indicate that females are more vulnerable to alcohol-related health risks including inflammation compared to males (White 2020). As such, our sex-dependent findings are clinically relevant, as they reinforce the biological vulnerability that females may have in comparison to males, and additionally indicate that ELS could be a larger risk factor for inflammation-driven alcohol use behaviors compared to males.

Limitations of this study include that the data were cross-sectional due to the parent study design. Mediation analyses in cross-sectional data may result in biased estimates, and causal inferences can therefore not be concluded (Maxwell and Cole 2007). Moreover, the DPDD outcome was assessed during the seven days prior to blood sample collection. Given that the alcohol use outcome preceded the inflammation composite mediator, temporal associations cannot be concluded from the present analyses. Sensitivity analyses examined the alternate temporal pathway of ELS predicting inflammation through recent alcohol use, and results revealed a null indirect effect. This null indirect effect in the alternate pathway, coupled with the significant indirect effect between ELS and alcohol use through inflammation in the main analyses, provide support that inflammation plays a role in linking ELS and alcohol use. However, future longitudinal analyses should be utilized to provide causal support that inflammation is a biological mechanism linking ELS to alcohol use. In addition, though cytokines and CRP have high overall temporal stability in health adults (Walsh et al. 2023; Wu et al. 2017) and likely would not have fluctuated significantly if assessed prior to the drinking outcome, future studies should utilize longitudinal study designs. In terms of acute fluctuations in inflammatory markers, physiological and environmental factors can cause acute changes in inflammation. Due to these acute fluctuations, future studies should consider using repeated blood measures, rather than a single blood draw, to better capture inflammation status. Moreover, future studies should consider controlling for additional covariates that may impact inflammatory markers such as recent sickness, medications, and smoking status. These potentially confounding variables were not included in the present analysis to conserve power in the mediation models. Therefore, the present study presents preliminary findings of peripheral inflammation mediating the relationship between ELS and alcohol use, and these findings should be replicated in a larger sample size.

In conclusion, this preliminary study identified that high levels of ELS are a sex-dependent risk factor for high levels of alcohol consumption through elevated peripheral inflammation in treatment-seeking individuals with moderate-to-severe AUD. ELS was seen to be a sex-dependent risk factor as the above pattern was more salient for females compared to males. These findings extend previous literature positing that inflammation mediates the relationship between ELS and alcohol use by (1) focusing on a sample of treatment-seeking individuals with moderate-to-severe AUD to increase clinical relevance of findings, (2) broadening measurement of inflammation to include inflammatory cytokines, and (3) considering the moderating role of sex in the relationship between ELS and inflammation. Identifying the role of peripheral inflammation in the link between ELS and alcohol consumption provides an underexplored treatment target for individuals with AUD who have experienced ELS, thereby advancing precision medicine. Specifically, these findings suggest that high-ELS individuals with AUD, particularly females, may be ideal candidates for treatments targeting the inflammatory system.

## Data Availability

Data are available upon reasonable request.

## Declarations

### Clinical trial registration

Study is a secondary analysis of a clinical trial (ClinicalTrials.gov identifier: NCT03594435, registered July 11, 2018).
